# Efficacy of infliximab and adalimumab therapy in very early onset, severe ulcerative colitis 

**Published:** 2021

**Authors:** Pejman Rohani, Hosein Alimadadi, Fatemeh Abdollah Gorji, Shabnam Shahrokh, Mohammad Reza Zali

**Affiliations:** 1 *Pediatric Gastroenterology and Hepatology Research Center, Children Center of Excellence, Children’ s Medical Center, Tehran University of Medical Sciences, Tehran, Iran*; 2 *Mofid children’s hospital, Clinical Research Development Center, Shahid Beheshti University of Medical Sciences, Tehran, Iran*; 3 * Gastroenterology and Liver Diseases Research Center, Research Institute for Gastroenterology and Liver Diseases, Shahid Beheshti University of Medical Sciences, Tehran, Iran*

**Keywords:** Infliximab, Adalimumab, Ulcerative colitis, Pediatric, Inflammatory bowel disease

## Abstract

**Aim:**

This multicenter study is the first one on Iranian children with very early onset ulcerative colitis (UC) and one of the few studies about the effect of biological therapy in children with UC under 7 years of age.

**Background:**

Children with very early onset inflammatory bowel disease (IBD) are diagnosed before 6 years of age

**Methods:**

The current study was performed on 14 children under 7 years of age with severe UC. Children with severe UC whose therapy with corticosteroid and azathioprine as conventional treatment had failed were treated with infliximab (IFX) and later with adalimumab (ADA).

**Results:**

Among the total 14 participants, 6 (43%) patients were female. Mean patient age was 4.9 years (range = 3–7 years), mean age at diagnosis was 3.4 years (range = 1.5–6 years), and mean duration of illness was 1.5 years. At the end of 54 weeks of therapy with IFX, 2 (14%) patients were in remission, 2 (14%) patients were mild, and 4 (29%) patients were moderate, with no secondary treatment failure (during the maintenance phase). A total of 6 (43%) patients had primary treatment failure (no response after 14 weeks of therapy). These patients were treated with ADA. At the end of 52 weeks of therapy, 3 (50%) of those 6 (100%) patients were referred for colectomy, 1 (17%) was in remission, and 2 (33%) patients had mild severity.

**Conclusion:**

The current study has shown that IFX is a safe and effective therapy for children with very early onset UC. ADA may be effective in the treatment of children with UC who are refractory to IFX.

## Introduction

 Children with very early onset inflammatory bowel disease (VEO-IBD) are diagnosed with IBD before 6 years of age. Monogenic defects of inborn error of immune disease are the probable cause of VEO-IBD in 15%–20% of these patients (1). Diagnosis of this subgroup of patients is the first priority of management. Other than the conventional treatment for monogenic immune disorders, such as corticosteroids and immunomodulators, biological therapy is recommended in refractory cases (2). The current multicenter study is the first of its kind on Iranian children with very early onset ulcerative colitis (UC) and one of the few studies in the world on the effect of biologic therapy in children with UC under 7 years of age. 

## Methods

**Figure 1:**
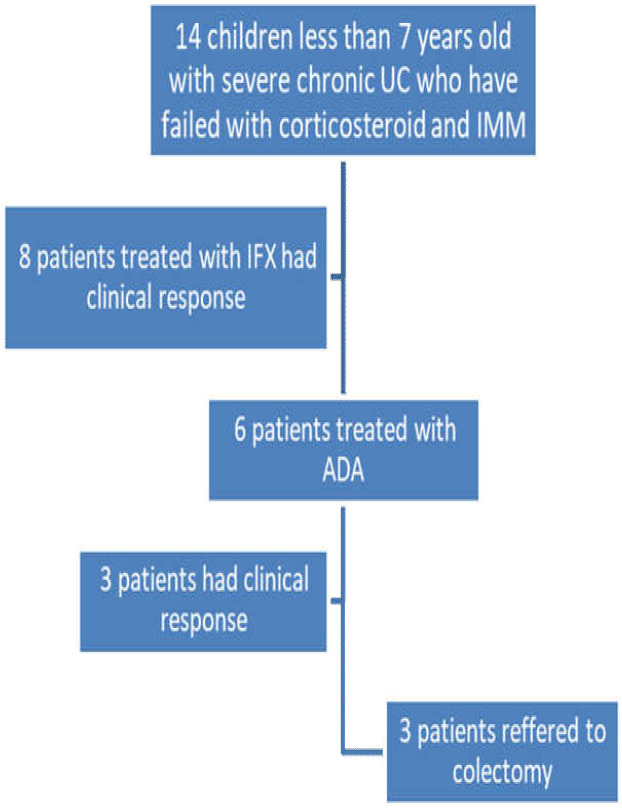
Method of study

This multicenter, retrospective cross-sectional study was performed on 14 children under 7 years of age with severe UC. Patients were admitted to Mofid Children’s Hospital or the Pediatric Center of Excellence between 2016 and 2019. All children with severe UC who had failed therapy with corticosteroid and azathioprine as conventional treatment were treated with infliximab (IFX) and later with adalimumab (ADA) if IFX therapy failed. UC was diagnosed based on clinical, radiological, and pathological findings. Thorough patient information was recorded with details about first clinical manifestations and changes during disease course and treatment; pediatric ulcerative colitis activity index (PUCAI); remission; partial response; primary treatment failure; lab data; radiological, endoscopic, and pathologic findings; and medication (supplementary file). Patients were studied for primary immune deficiency with an immunoglobulin panel (IgG, IgE, IgM, IgA), CD markers, nitroblue tetrazolium, and lymphocyte transformation test. All patients had formerly been treated with conventional therapy (corticosteroid and azathioprine). All candidates for biological therapy were revaluated for tuberculosis, hepatitis B, and hepatitis C. Written informed consent was obtained from parents before biological therapy was administered. Exclusion criteria included patients presenting with acute severe colitis, children with classic immunodeficiency, any clinical behavior like perianal disease or fistula formation or pathological findings like granuloma in favor of Crohn’s disease, and patients with poor compliance or irregular follow-up. The PUCAI was calculated every 2–8 weeks. Adverse drug reactions were monitored during the treatment course. The disease was considered in remission if PUCAI < 10; a change of at least 20 points from baseline was defined as partial response. Primary treatment failure was defined as no response seen in 14 weeks post-therapy. Treatment protocol was induction with intravenous IFX (10 mg/kg) first, then at 2- and 6-week intervals. Maintenance therapy was administered every 8 weeks. Patients who responded to treatment were followed for 54 weeks. Those children who were unresponsive to IFX were switched to ADA. Treatment protocol was induction with subcutaneous ADA (80 mg) first, then 40 mg 2 weeks later. In maintenance therapy, ADA (20 mg) was administered every other week. Patients who responded to therapy were followed for 52 weeks. Research data was analyzed by SPSS version 25. Statistical analysis was conducted by Student’s *t*-test, Fisher’s exact test, ² test, repeated measurements, and logistic regression. The method of study is summarized in Figure 1.

**Ethical Considerations**

This study was approved by the Ethics Committee of the Research Institute of Children Health in Shahid Beheshti University of Medical Sciences. The committee did not accept ADA escalation therapy in children under 7 years of age. Written informed consent was obtained from parents of children for treatment with IFX and ADA. Permission for colectomy was not accepted by 2 families during treatment with ADA after 6–8 weeks of therapy. However, eventually, both patients underwent colectomy with family permission. 

## Results

Out of a total of 14 patients, 6 (43%) were female. Patient mean age at the beginning of the study was 4.9 years (3–7 years) and at diagnosis was 3.4 years (1.5–6 years). Mean duration of illness was 1.5 years. Disease extent according to the Paris classification was E1 = 2 (14%), E2 = 3 (21%), E3 = 3 (29%), and E4 = 4 (36%). Mean value of PUCAI at the time of induction for patients who had received IFX and those who had received ADA was 74.8 ± 6.8 and 71.8 ± 6.6, respectively. No significant relationship was detected between any of the demographic data and initial treatment failure, clinical response, or remission. Family history of IBD was negative in all 14 patients. The details of study data of patients are given in Table 1.

**Table 1:** Study data

Subject	Total (N=14)		Infliximab (N=8)*	Adalimumab (N=6)†
Age mean (range) y	4.9 (3-7)		5.2	4.6
Age at diagnosis mean (range)y	3.4 (1.5-6)		3.5	3.3
Sex	Male 8 (57%) Female 6 (43%)		4 4	4 2
Disease duration (range)y	Mean 1.5 (1-3) Median 1.5			
Disease extent				
E1 E2 E3 E4	2 (14%) 3 (21%) 4(29%) 5(36%)		2 2 2 2	0 1 2 3
Infusion	212(mean=15.1)††		72(mean= 9)	116(mean=19.3)
Adverse drug reaction				
Skin reaction Acute Infusion Reaction Serum sickness like reaction Major infection Minor infection Flu like syndrome Other serious disease (malignancy, lupus, hepatitis)			3 14 0 0 5 1 0	0 10 0 0 2 0 0
Previous medication				
Azathioprine		14(100%)	8(100%)	6(100%)
5 ASA		4(28.5%)	3(21.5%)	1(17%)
Corticosteroid		14(100%)	8(100%)	6(100%)
Corticosteroid free (at the end of 52-54 weeks)		7(50%)	4(50%)	3(50%)
PUCAI (before treatment)		-	74.8±6.8	71.8±6.6
PUCAI (after treatment)		-	40.4±18.8	25.3±19.9
Colectomy rate		3(21.5%)	-	-

*Duration of therapy was 54 weeks in IFX group; † Duration of therapy was 52 weeks in ADA group; †† Each one of 6 patients in ADA group had received 4 doses of IFX.

**Therapy with IFX**

A change in PUCAI during the course of treatment and disease severity changes during treatment in patients under IFX therapy is illustrated in Figures 2A and 2B, respectively. After 8 weeks of treatment, 8 (57%) patients showed improvement, and the disease severity changed from severe to moderate. At the 14th week of therapy, one patient improved from moderate to mild. Those who responded to IFX were followed for 54 weeks. None of the patients lost response or had any severe side effects in holding IFX. At the end of 54 weeks, 2 (14%) patients were in remission, 2 (14%) patients were mild, and 4 (29%) patients were moderate, with no secondary treatment failure (during the maintenance phase). A total of 6 (43%) patients had primary treatment failure (no response after 14 weeks of therapy). These patients were recommended for colectomy. All parents had denied the colectomy. Then, patients were switched to ADA.

**Therapy with ADA**

A change in PUCAI during the course of treatment and disease severity changes during treatment in patients under ADA therapy are illustrated in Figures 3A and 3B, respectively. After 2 weeks of therapy, 2 (33%) patients had no improvement and, at the end of 6–8 weeks of therapy, were referred to the pediatric surgery team for colectomy. A total of 4 (67%) patients had partial response after 2 weeks of induction, and disease severity changed from severe to moderate. At the end of 26 weeks of therapy, 1 patient had improvement and the severity changed from moderate to mild, but 3 other patients had no improvement. These 3 patients were referred to the pediatric surgery ward, but parents did not accept colectomy as a treatment option. Continuation with medical therapy was the only choice. As treatment continued, one patient’s condition changed from mild to remission, and one patient worsened and was referred for colectomy. Two patients with moderate severity had improvement and changed to mild. At the end of 52 weeks of therapy, 3 (50%) patients out of 6 (100%) were referred for colectomy, one (17%) patient was in remission, and two (33%) patients had changed to mild severity.

**Figure 2A:**
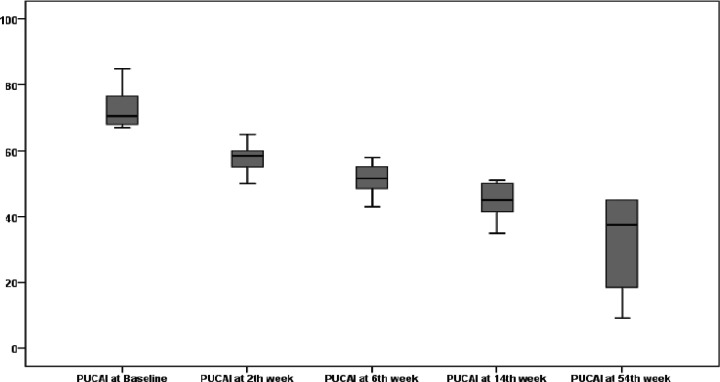
PUCAI changes during treatment with infliximab

**Figure 2B:**
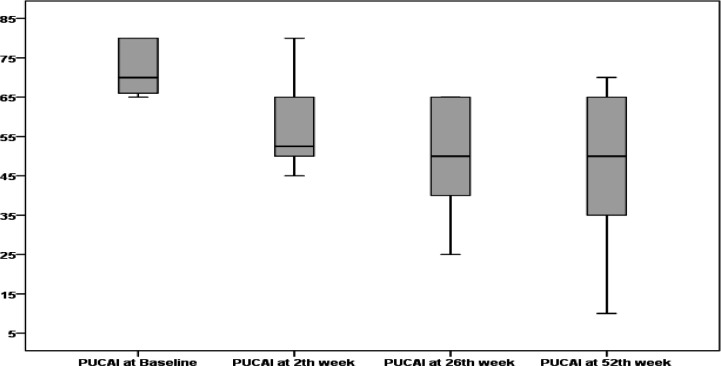
Severity changes of disease during treatment with infliximab

## Discussion

**Figure 3A:**
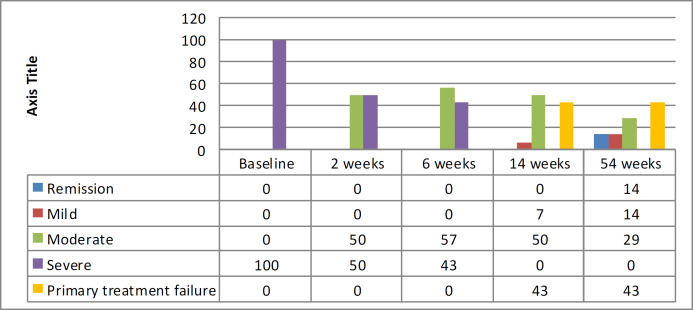
PUCAI changes during treatment with adalimumab

**Figure 3B:**
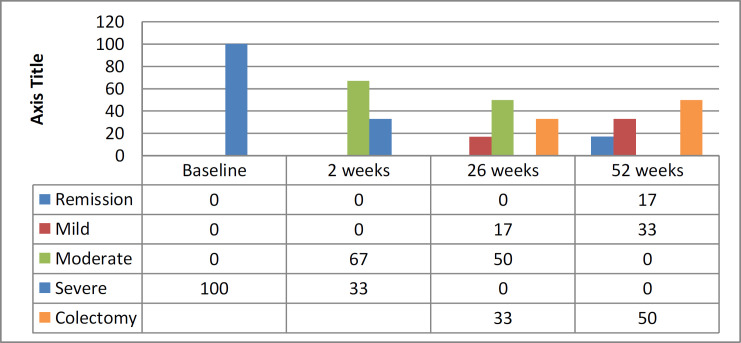
Severity changes of disease during treatment with adalimumab

VEO-IBD (disease beginning before 6 years of age) is found in 6%–15% of children with IBD. Approximately 15%–20% of children with VEO-IBD have a monogenic type of disease with some kind of genetic defect predisposing patients to immune dysregulation (1). The rest of the children have adult-like behavior with a polygenic defect. These patients are suitable candidates for conventional treatment such as corticosteroids or immunomodulators. Biological therapy is an alternative therapy in situations like failure of conventional treatment or severe intensity at onset of disease. In 2011, IFX was approved by the FDA for the treatment of children above 6 years of age with moderate to severe UC (2, 3). IFX administration or other biological therapies like ADA are not approved for routine administration in the treatment of children under 6 years of age. The current research is one of the few studies on children with UC under 7 years of age. In addition, studies about ADA in children with UC, especially in children under 7 years of age, are very limited.

According to the latest ESPGHAN guidelines on the management of children with UC, IFX has induced remission in 64% of pediatric patients above 7 years of age. Colectomy rate was 39% at the end of 2 years. Corticosteroid freeness was 38% at the end of 1 year and 21% at the end of 2 years of follow-up (3). After 14 weeks of therapy, 43% of patients in the current study had primary treatment failure. No patients were in remission. Only 1 patient (7%) was mild, and 7 patients (50%) were in moderate severity. A total of 57% of patients continued therapy for 54 weeks. At that time, 14% (2 patients) were in remission, 14% (2 patients) were mild, and 28% (4 patients) had moderate severity. Corticosteroid freeness was 50% at the end of 1 year. 

According to Bramuzzo et al., 32% (9/28) of patients with VEO-UC after 14 weeks of IFX therapy were in remission. A total of 25% (7/28) of patients with VEO-UC continued therapy until 54 weeks. A total of 28.5% (2/7) of patients who continued therapy were in remission. In total, only 7% (2 of 28) of pediatric patients with VEO-UC were in remission at the end of 54 weeks (4). 

One important difference between Bramuzzo et al.’s study and the current one is dose escalation therapy. Bramuzzo et al. started with 5 mg/kg and then escalated therapy to 10 mg/kg in accordance with standard guidelines. In the current study, treatment began with 10 mg/kg of IFX because of limitations in measuring serum drug levels and the worse condition of the current patients. In addition, ADA was not an approved therapy for the treatment of UC, especially in children under 7 years of age. The better response of the current patients might be due to the higher dose of IFX in the induction phase. According to a study by Kelsen et al., the corticosteroid freeness effect of IFX at the end of 1 year was 25% (12 patients) in patients with VEO-IBD, but IBD subclassification was not performed in their study. In addition, 36% of patients tolerated treatment for 1 year (5). A new study by Assa et al. on 4 infants with VEO-IBD has shown that therapeutic drug monitoring may be the most effective means of deciding about the dose or about escalation of therapy. To induce remission in children with VEO-IBD, 22 mg/kg may be needed (6). Many other studies with reasoning about different pharmacokinetic properties of IFX between children of different ages and BMIs have concluded that therapeutic drug monitoring is a very sensitive way of regulating the dose or interval of injection (7).

According to the latest ESPGHAN guidelines, ADA should be considered only in children above 6 years of age who lost response to IFX. Then, administration is off-label in children below 7 years of age and in patients who are refractory to IFX (3). SIGENP-IBD Registry data provides information on a study conducted by Aloi et al. on 32 UC patients above 6 years of age. All had a history of treatment with IFX. A total of 41% of patients were in remission, and 28% of patients had mucosal healing after 52 weeks of therapy. No difference was found between nonresponders and patients intolerant to ADA (8). A study by Steiner et al. on 133 children with UC below 18 years of age showed that 63% and 68% of patients achieved clinical remission after 12 and 24 months of therapy, respectively. Patients were probably biologically naïve (9). Volonaki et al. studied 11 patients with UC who were refractory to IFX. After 24 months of therapy, 54% had clinical remission and 36% underwent colectomy (10). Wiernicka et al. studied the efficacy of ADA on 6 children (above 6 years of age) with UC. Three patients were refractory to IFX. After 12 months of therapy, 50% (3/6) had clinical remission. At the end of 8 weeks of therapy, 2 out of 6 were in remission (11). In a study by Noe et al., 2 out of 3 children with UC had improvement in the Lichtiger Colitis Activity Index (LCAI) score after 6 months of therapy. All children were refractory to IFX (12). In another study by Romagnoli et al. on 4 children with UC above 6 years of age, all were clinically responsive after 6 months of therapy (13).

The current study has shown that even in children below 7 years of age, ADA may prevent colectomy in 50% of children who are refractory to IFX. Out of 6 patients who were treated with ADA for 52 weeks, 3 patients had improvement. A total of 4 patients had improvement just after 2 weeks from the start of therapy. 

After a treatment course of only 26 weeks, the condition of one patient changed from moderate to mild. Continuation of therapy for 52 weeks led to a change in severity, and at the end, one patient was in remission (17%), one patient was referred for colectomy, and 2 patients had improvement with mild severity. Corticosteroid freeness was 50% at the end of 1 year. 

Total colectomy rate after 24 months of biological therapy was 21.5%. No life-threatening or severe adverse reaction was seen after 216 infusions or injections of IFX or ADA. Advantages the current study has over others are the age of the children (below 7 years) and the type of disease (UC). In addition, the current study evaluated the efficacy of ADA in children who are refractory to IFX. Most studies have assessed the efficacy of ADA in children who lost response to IFX. Overall, studies on biological therapy in children with early onset IBD are limited. 

The current study has some important limitations. First, a small sample size confines the study results. Second, off-label usage of ADA in children below 7 years of age led the ethics committee of our university to hold escalation of ADA to 40 mg in refractory cases. Third, it was not possible to measure serum drug monitoring or antibody formation at predefined intervals. Fourth, the current study evaluated clinical remission only. Most parents of patients in this study did not permit recolonoscopy, due to which evaluation of mucosal healing was not possible. In addition, all 6 patients who were treated with ADA were not naive, and all had treatment courses with IFX.

The current study has shown that IFX and ADA are safe and effective therapies in children with VEO-UC. In addition, ADA may be effective in the treatment of children who are refractory to IFX. Larger multicenter studies across countries on children with VEO-IBD are required to confirm the efficacy of these medications.

## Conflict of interests

The authors declare that they have no conflict of interest.
